# Which Factors Are Associated With Reproductive Outcomes of DOR Patients in ART Cycles: An Eight-Year Retrospective Study

**DOI:** 10.3389/fendo.2022.796199

**Published:** 2022-06-23

**Authors:** Lu Li, Bo Sun, Fang Wang, Yile Zhang, Yingpu Sun

**Affiliations:** ^1^ Center for Reproductive Medicine, The First Affiliated Hospital of Zhengzhou University, Zhengzhou, China; ^2^ Henan Key Laboratory of Reproduction and Genetics, The First Affiliated Hospital of Zhengzhou University, Zhengzhou, China; ^3^ Henan Provincial Obstetrical and Gynecological Diseases (Reproductive Medicine) Clinical Research Center, The First Affiliated Hospital of Zhengzhou University, Zhengzhou, China; ^4^ Henan Engineering Laboratory of Preimplantation Genetic Diagnosis and Screening, The First Affiliated Hospital of Zhengzhou University, Zhengzhou, China

**Keywords:** IVF *in vitro* fertilization, AMH (anti-Müllerian hormone), DOR (diminished ovarian reserve), reproductive outcomes, antral follicle count (AFC)

## Abstract

**Introduction:**

Women with diminished ovarian reserve (DOR) have a lower pregnancy rate and higher cancellation rate compared to those without DOR when seeking assisted reproductive technology. However, which factors are associated with reproductive outcomes and whether AMH is a predictor of clinical pregnancy remain unclear.

**Objective:**

This retrospective study was designed to find factors associated with reproductive outcomes in DOR patients and then discuss the role of AMH in predicting cycle results among this population.

**Method:**

A total of 900 women were included in the study. They were diagnosed with DOR with the following criteria: (i) FSH > 10 IU/L; (ii)AMH < 1.1 ng/ml; and (iii) AFC <7. They were divided into different groups: firstly, based on whether they were clinically pregnant or not, pregnant group vs. non-pregnant group (comparison 1); secondly, if patients had transferrable embryos (TE) or not, TE vs. no TE group (comparison 2); thirdly, patients undergoing embryo transfer (ET) cycles were divided into pregnant I and non-pregnant I group (comparison 3). The baseline and ovarian stimulation characteristics of these women in their first IVF/ICSI cycles were analyzed. Logistic regression was performed to find factors associated with clinical pregnancy.

**Results:**

Of the 900 DOR patients, 138 women got pregnant in their first IVF/ICSI cycles while the rest did not. AMH was an independent predictor of TE after adjusting for confounding factors (adjusted OR:11.848, 95% CI: 6.21-22.62, P< 0.001). Further ROC (receiver operating characteristic) analysis was performed and the corresponding AUC (the area under the curve) was 0.679 (95% CI: 0.639-0.72, P< 0.001). Notably, an AMH level of 0.355 had a sensitivity of 62.6% and specificity of 65.6%. However, there was no statistical difference in AMH level in comparison 3, and multivariate logistic regression showed female age was associated with clinical pregnancy in ET cycles and women who were under 35 years old were more likely to be pregnant compared to those older than 40 years old (adjusted OR:4.755, 95% CI: 2.81-8.04, P< 0.001).

**Conclusion:**

AMH is highly related to oocyte collection rate and TE rate,and 0.355 ng/ml was a cutoff value for the prediction of TE. For DOR patients who had an embryo transferred, AMH is not associated with clinical pregnancy while female age is an independent risk factor for it.

## Background

Diminished ovarian reserve (DOR) refers to the reduction of the quantity of oocytes in the ovary, which is one of the major causes of infertility in women of child-bearing age (1). Ovarian surgery and gene mutation may be associated with DOR while most patients with DOR cannot find an identified etiology (2, 3). Patients with DOR have a lower number of oocytes acquired and a rate of high-quality embryos compared to those with normal ovarian reserve (NOR), and the rate of a clinical pregnancy is lower while the early miscarriage rate is higher (4–6). Although various treatments are made to assist them to improve the outcome of pregnancy, it remains a big challenge for clinicians.

Based on the Bologna criteria (7), follicle-stimulating hormone (FSH), anti-mullerian hormone (AMH), and antra follicular count (AFC) are the most frequently used biomarkers to access the ovarian reserve, and the latter two have gained widespread attention in recent years. Generally, AMH levels and AFC decline while the incidence of DOR increases, however, discordance between AFC and AMH levels is not rare in clinical work. Measuring by ultrasound, AFC is highly affected by different machines and operating doctors. Studies have demonstrated that priority should be given to AMH compared to AFC in predicting ovarian marker and fertility (8–10).

Controversy has existed on whether AMH is associated with reproductive outcomes in assisted reproductive technology (ART) cycles. A meta-analysis reviewed 19 articles including unspecified ovarian reserve, DOR, and polycystic ovary syndrome (PCOS) patients. Results showed that AMH had weak association with clinical pregnancy but could be a predictor in DOR women (11). A study analyzed 85,062 fresh and embryo-thawed (ET) cycles and demonstrated AMH cannot be a reliable independent predictor of live birth rate (12).

In this retrospective study, we collected data from the first IVF/ICSI cycles of patients with DOR and analyzed baseline and controlled ovarian stimulation (COS) characteristics to find out factors associated with reproductive outcomes and then discuss the role of AMH in predicting cycle results among this population.

## Methods and Materials

### Patient Selection

**Figure 1:**
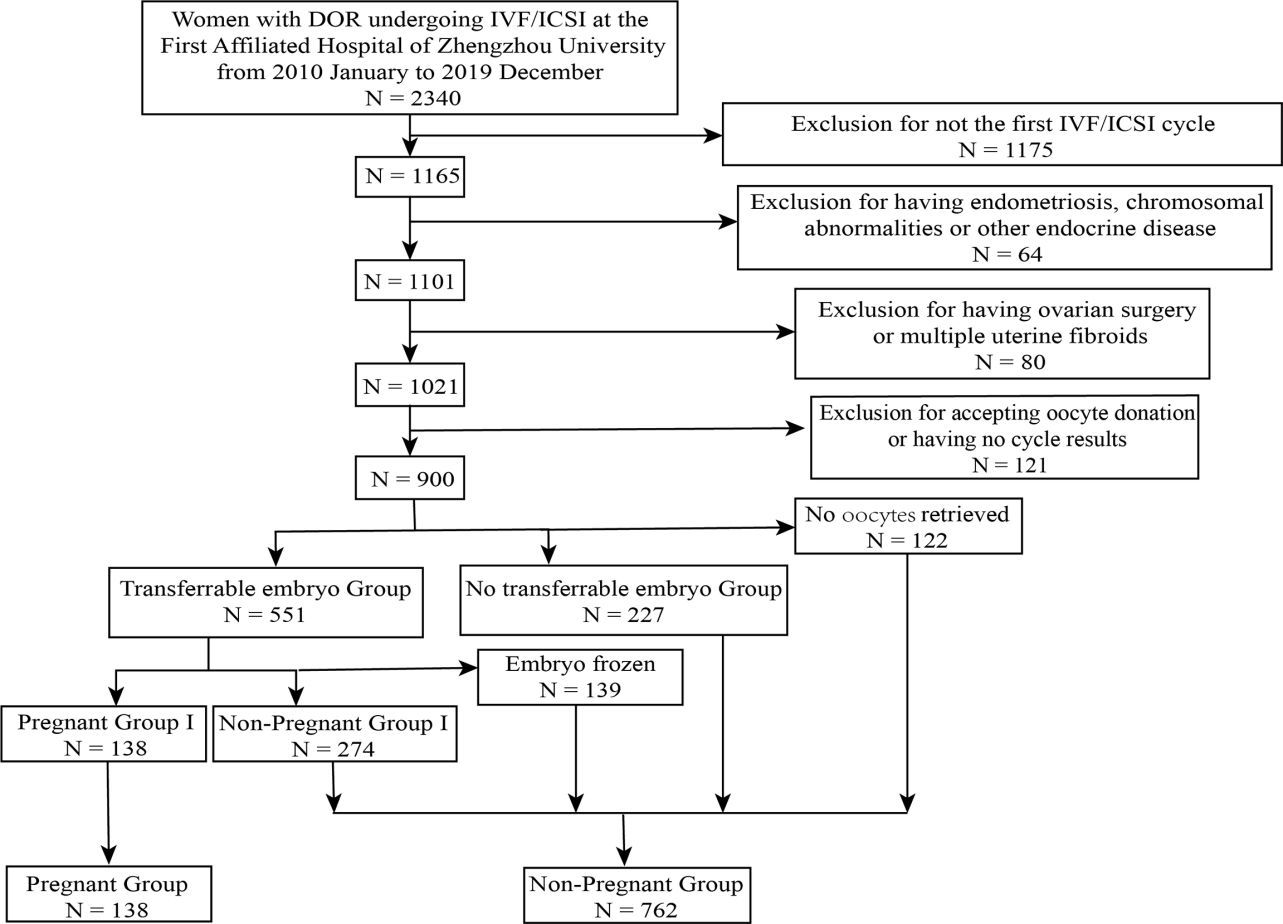
Flow chart of patient selection.

Patients who came to the First Affiliated Hospital of Zhengzhou University for autologous IVF/ICSI cycles were enrolled into this study during January 2011 to December 2019. Patients who have fulfilled the following criteria were included: (i) FSH > 10 IU/L and AMH < 1.1 ng/ml and AFC <7; (ii) the first fresh IVF/ICSI cycle in our center. While the participants who had (i) endometriosis, polycystic ovarian syndrome (PCOS); (ii) chromosomal abnormalities; (iii) hypertension, diabetes, or other chronic diseases; (iv) immune system diseases, such as hypothyroidism; (v) multiple uterine fibroids or a history of ovarian surgery or chemotherapy or radiation exposure; (vi) experienced IVF/ICSI cycles at other hospitals; (vii) premature ovarian insufficiency were excluded (**Figure 1**) as our purpose was to characterize women with idiopathic decrease in ovarian reserve and eliminate other possible confounding factors that had influence on reproductive outcomes. This study was performed under institutional review board approval.

### Grouping Method

A total of 900 patients with DOR were included (**Figure 1**). To find out factors associated with reproductive outcomes in DOR patients at their first ART cycles, firstly, patients were divided into two groups based on whether they were clinically pregnant or not: pregnant group vs. non-pregnant group (comparison 1); secondly, patients who had transferrable embryos (TE) were compared with those did not: TE vs. no TE group (comparison 2); thirdly, patients undergoing embryo transfer (ET) cycles were divided into two groups: pregnant I and non-pregnant I group (comparison 3). Clinical pregnancy was defined as the following: 35 days after transplantation, transvaginal ultrasound examination showed that there was at least one gestational sac in the uterus, including ectopic pregnancy (13).

### AMH Level Detection

Two ml blood samples were aseptically collected from the subjects on days 2 – 4 of the menstrual cycle to assess basal AMH. After centrifugation, serum was analyzed by an electrochemical luminescence analyzer (Roche, Cobas e601, Canada) to detect AMH levels (ng/ml). The theoretical sensitivity of the method was 0.006 ng/ml. Within batches and between batches coefficient of variations were ≤10% and ≤15%, respectively.

### Controlled Ovarian Stimulation Protocol

The ovarian stimulation protocol was determined by the ovarian reserve testing (AMH, AFC and basal FSH) of each patient.

#### Progestin-Primed Ovarian Stimulation Protocol (PPOS)

Medroxyprogesterone acetate and human menopausal gonadotropin (HMG) were used on the third day of menstruation, human chorionic gonadotropin (HCG) was used at the time of triggering.

#### Follicular Phase Long*-*Acting Protocol

Patients were given a starting dose of 3.75 mg GnRH agonist (GnRH-a) on the second day of menstruation. Gonadotropins (Gn) were used to induce ovulation, and we adjusted the dose according to the number, size, and growth of the follicles.

#### Mild Stimulation Protocol

Letrozole was given to patients at a dose of 2.5 mg per day on the third day of menstruation, and HMG was added on the fifth day. Once the diameter of a primary follicle was > 18 mm, HCG and Gn were used.

#### Natural Cycle

The number, size, and growth of follicles and hormone levels, especially LH, E2, and P4, were observed during menstruation to determine the time of triggering.

#### Luteal Phase Short*-*Acting Long Protocol

GnRH-a was used on the 21st day of menstruation, and ultrasound and hormone levels were used to observe the growth of the follicles. Ovulation was induced using HCG according to the size of the follicles.

#### GnRH Antagonist Protocol

FSH was given to patients on their second day of menstruation, and Gn and HCG were injected when the diameter of the primary follicle was > 18 mm.

In the above protocols, HCG was used for 36-37 h before oocyte retrieval. IVF or ICSI was used according to the semen quality of the husband.

### Statistical Methods

The baseline and ovarian stimulation characteristics of patients were compared between each two groups (the grouping method was as described above). Continuous variables were compared by Mann–Whitney U since they were not normally distributed. Chi-squared tests were used to compare categorical variables. The numerical data are presented as the mean with standard deviation (SD), while categorical variables are shown as % (n/N). We performed the univariate and multivariate logistic regression analyses to examine factors that were associated with reproductive outcomes. IBM SPSS version 26.0 (IBM Corp., Armonk, NY, USA) was used, and a P value < 0.05 was considered statistically significant.

## Results

Of the 900 patients who met the criteria in the study, 778 (86.44%) women had oocytes retrieved at their first cycle, 551 (61.22%) women had transferrable embryos after egg collection, 139 (15.44%) patients had embryos frozen, and 138 (15.33%) women got pregnant after implantation.

### Baseline Characteristics

As shown in **Table 1**, in comparison 1, both women and their husbands in the pregnant group were younger than those in the non-pregnant group (34.64 ± 0.43 vs. 38.16 ± 0.22, P < 0.001; 35.13 ± 0.51 vs. 38.80 ± 0.24, P < 0.001). Compared to the non-pregnant group, patients in the pregnant group had shorter years of infertility (4.14 ± 0.31 vs. 5.02 ± 0.16, P=0.045) and experienced fewer times of delivery (0.47 ± 0.05 vs. 0.63 ± 0.02, P =0.010); their basal follicle-stimulating hormone (FSH) and luteinizing hormone (LH) levels were much lower (15.23 ± 0.58 vs. 17.46 ± 0.36, P =0.006; 6.17 ± 0.33 vs. 8.10 ± 0.28, P = 0.003) while basal AMH levels and AFC were much higher (0.54 ± 0.02 vs. 0.37 ± 0.01, P< 0.001; 3.70 ± 0.14 VS 2.79 ± 0.07, P< 0.001). There was no significant difference in the type of infertility, number of pregnancy or abortion, body mass index (BMI), basal estradiol (E2), progesterone (P4), testosterone (T) levels, or basal endometrial thickness between two groups.

**Table 1:** Baseline characteristics and hormonal profiles between groups of women with DOR undergoing IVF/ICSI.

	Comparison 1			Comparison 2			Comparison 3		
	Pregnant	Non-pregnant	p value	TE	No TE	p value	Pregnant I	Non-pregnant I	p value
Number	138	762		551	227		138	274	
Female age	34.64 ± 0.43	38.16 ± 0.22	<0.001	37.30 ± 0.24	38.09 ± 0.42	0.021	34.64 ± 0.43	38.13 ± 0.29	<0.001
Male age	35.13 ± 0.51	38.80 ± 0.24	<0.001	37.99 ± 0.27	38.97 ± 0.46	0.055	35.13 ± 0.51	39.07 ± 0.36	<0.001
Type of infertility			0.100			0.612			0.020
Primary infertility	51 (36.96)	228 (29.92)		167 (30.31)	73 (32.16)		51 (36.96)	71 (25.91)	
Secondary infertility	87 (63.04)	534 (70.08)		384 (69.69)	154 (67.84)		87 (63.04)	203 (74.09)	
Years of infertility	4.14 ± 0.31	5.02 ± 0.16	0.045	4.80 ± 0.19	5.04 ± 0.30	0.350	4.14 ± 0.31	5.13 ± 0.29	0.086
No. of previous pregnancy	1.23 ± 0.10	1.40 ± 0.04	0.126	1.36 ± 0.49	1.36 ± 0.08	0.875	1.23 ± 0.10	1.49 ± 0.07	0.024
No. of previous deliveries	0.47 ± 0.05	0.63 ± 0.02	0.010	0.59 ± 0.03	0.62 ± 0.04	0.573	0.47 ± 0.05	0.66 ± 0.04	0.005
No. of previous abortion	0.49 ± 0.06	0.59 ± 0.03	0.149	0.56 ± 0.03	0.59 ± 0.05	0.723	0.49 ± 0.06	0.62 ± 0.04	0.083
BMI	22.86 ± 0.24	23.07 ± 0.10	0.506	23.01 ± 0.12	23.24 ± 0.20	0.644	22.86 ± 0.24	23.04 ± 0.17	0.544
Basal FSH (mIU/mL)	15.23 ± 0.58	17.46 ± 0.36	0.006	15.79 ± 0.33	18.00 ± 0.72	0.001	15.23 ± 0.58	14.25 ± 0.29	0.155
Basal E2 (pg/mL)	38.09 ± 3.76	80.48 ± 9.80	0.177	73.10 ± 12.60	77.19 ± 11.52	0.682	38.09 ± 3.76	93.98 ± 23.76	0.219
Basal P4 (ng/mL)	0.45 ± 0.03	0.46 ± 0.01	0.215	0.45 ± 0.01	0.46 ± 0.02	0.213	0.45 ± 0.03	0.45 ± 0.02	0.227
Basal LH (mIU/mL)	6.17 ± 0.33	8.10 ± 0.28	0.003	7.19 ± 0.28	7.67 ± 0.47	0.295	6.17 ± 0.33	6.55 ± 0.39	0.980
Basal T (ng/mL)	0.20 ± 0.01	0.19 ± 0.00	0.927	0.20 ± 0.01	0.19 ± 0.01	0.345	0.20 ± 0.01	0.19 ± 0.01	0.968
AMH (ng/mL)	0.54 ± 0.02	0.37 ± 0.01	<0.001	0.48 ± 0.01	0.30 ± 0.02	<0.001	0.54 ± 0.02	0.53 ± 0.02	0.677
Antral follicular count	3.70 ± 0.14	2.79 ± 0.07	<0.001	3.18 ± 0.08	2.72 ± 0.12	0.002	3.70 ± 0.14	3.33 ± 0.11	0.049
Basal endometrial thickness	6.21 ± 0.331	6.24 ± 0.13	0.764	6.32 ± 0.15	6.07 ± 0.23	0.591	6.21 ± 0.33	6.65 ± 0.21	0.205
Live birth*	96 (10.67)	/							
Abortion*	38 (4.22)	/							
Ectopic pregnancy*	4 (0.44)	/							
Transferrable embryos*	138 (15.33)	227 (25.22)							
Embryo frozen*	0 (0.00)	139 (15.44)							

Data are mean ± standard deviation or N (% of response group * % of all participants). FSH, follicle-stimulating hormone; E2, estradiol; P4, progesterone; LH, luteinizing hormone; T, testosterone; BMI, body mass index; AMH, anti-Mullerian hormone; TE, transferrable embryo.

In terms of transferrable embryo, only age of female (37.30 ± 0.24 vs. 38.09 ± 0.42, P = 0.021), basal FSH levels (15.79 ± 0.33 vs. 18.00 ± 0.72, P = 0.001), basal AMH levels (0.48 ± 0.01 vs. 0.30 ± 0.02, P < 0.001), and AFC (3.18 ± 0.08 vs. 2.72 ± 0.12, P = 0.002) differed between TE and no TE group. Others were not significantly different.

In comparison 3, FSH (15.23 ± 0.58 vs. 14.25 ± 0.29, P = 0.155) and AMH (0.54 ± 0.02 vs. 0.53 ± 0.02, P = 0.68) had no statistical difference between pregnant I and non-pregnant I group, while AFC (3.70 ± 0.14 vs. 3.33 ± 0.11, P = 0.049) was at the threshold value of P < 0.05. Both maternal (34.64 ± 0.43 vs. 38.13 ± 0.29, P < 0.001) and paternal (35.13 ± 0.24 vs. 39.07 ± 0.36, P< 0.001) age differed significantly between the two groups.

### Ovarian Stimulation Characteristics

As shown in **Table 2**, patients in the pregnant group used more gonadotropin (Gn) and the length of stimulation was much longer than those in the non-pregnant group. In addition, their E2 levels on the day of HCG administration was much higher (1673.67 ± 92.12 vs. 1036.99 ± 32.80, P< 0.001) and the endometrial thickness on that day was much thicker (11.57 ± 0.23 vs. 10.05 ± 0.11, P< 0.001). Not surprisingly, pregnant women had more oocytes retrieved (5.11 ± 0.26 vs. 3.11 ± 0.10, P< 0.001) and had more embryos to implant (2.70 ± 0.14 vs. 1.82 ± 0.07, P< 0.001) compared to women who were not pregnant. Women who were pregnant were more likely to be treated with follicular phase long-acting protocol (74/138 53.62%) and luteal phase ultra-long protocol (51/138 36.96%), however, there was no significant difference in the embryo stage when transferring between two groups.

**Table 2:** Ovarian stimulation characteristics between groups of women with DOR undergoing IVF/ICSI.

	Comparison 1			Comparison 2			Comparison 3		
	Pregnant	Non-pregnant	p value	TE	No TE	p value	Pregnant I	None-pregnant I	p value
Number	138	762		551	227		138	274	
Total amount of Gn (IU)	3823.46 ± 82.49	3039.97 ± 50.26	<0.001	3524.21 ± 50.20	2958.22 ± 89.92	<0.001	3823.46 ± 82.49	3828.10 ± 59.99	0.767
Duration of stimulation (d)	13.23 ± 0.24	10.95 ± 0.15	<0.001	12.30 ± 0.15	10.68 ± 0.27	<0.001	13.23 ± 0.24	13.09 ± 0.19	0.453
Endometrial thickness on HCG (mm)	11.53 ± 0.23	10.23 ± 0.13	<0.001	10.96 ± 0.12	9.93 ± 0.20	<0.001	11.57 ± 0.23	11.48 ± 0.15	0.904
Hormone levels on HCG									
E2 (pg/mL)	1673.67 ± 92.12	1036.99 ± 32.80	<0.001	1435.10 ± 44.88	768.02 ± 37.21	<0.001	1673.67 ± 92.12	1579.76 ± 64.24	0.326
LH (mIU/mL)	5.85 ± 0.33	8.21 ± 0.69	<0.001	3.36 ± 0.18	7.47 ± 0.57	<0.001	1.95 ± 0.19	2.32 ± 0.14	0.070
P4 (ng/mL)	0.63 ± 0.04	0.61 ± 0.03	0.293	0.67 ± 0.04	0.53 ± 0.05	0.001	0.63 ± 0.04	0.75 ± 0.07	0.483
No. of ≧14mm oocytes	4.32 ± 0.31	3.20 ± 0.17	<0.001	3.42 ± 0.10	1.81 ± 0.09	<0.001	4.11 ± 0.20	3.73 ± 0.13	0.098
Total oocytes retrieved	5.86 ± 0.48	3.90 ± 0.23	<0.001	4.06 ± 2.91	2.02 ± 1.84	<0.001	5.11 ± 0.26	4.26 ± 0.16	0.004
Rate of MII oocytes	5.02 ± 0.41	3.29 ± 0.20	<0.001	3.41 ± 2.55	1.34 ± 1.31	<0.001	4.38 ± 0.23	3.54 ± 0.14	0.001
Rate of 2PN embryos	3.86 ± 0.32	2.54 ± 0.15	<0.001	2.72 ± 1.94	0.66 ± 1.02	<0.001	3.55 ± 0.19	2.78 ± 0.11	<0.001
No. of transferrable embryo	2.79 ± 0.18	1.87 ± 0.09	<0.001	2.04 ± 1.24	0		2.68 ± 0.12	2.05 ± 0.07	<0.001
No. of good-quality embryos	2.54 ± 0.17	1.72 ± 0.09	<0.001	/	/		2.39 ± 0.12	1.80 ± 0.07	<0.001
No. of embryo transferred	1.68 ± 0.07	0.95 ± 0.06	<0.001	/	/		1.76 ± 0.04	1.59 ± 0.03	0.001
Stimulation protocol			<0.001			<0.001			0.010
PPOS	0 (0)	75 (9.84)		25 (4.54)	18 (7.93)		0 (0)	0 (0)	
Follicular phase long-acting protocol	74 (53.62)	188 (24.67)		214 (38.84)	45 (19.82)		74 (53.62)	124 (45.26)	
GnRH antagonist protocol	12 (8.70)	233 (30.58)		133 (24.14)	79 (34.80)		12 (8.70)	54 (19.71)	
Mild stimulation protocol	1 (0.72)	81 (10.63)		20 (3.63)	30 (13.22)		1 (0.72)	0 (0)	
Luteal phase short-acting long protocol	51 (36.96)	169 (22.18)		157 (28.49)	50 (22.03)		51 (36.96)	96 (35.04)	
Natural cycle	0 (0)	16 (2.10)		2 (0.36)	5 (2.20)		0 (0)	0 (0)	
Type of ART			0.005			0.005			0.881
IVF	113 (81.88)	686 (90.03)		469 (85.12)	210 (92.51)		113 (81.88)	226 (82.48)	
ICSI	25 (15.22)	76 (9.97)		82 (14.88)	17 (7.49)		25 (18.12)	48 (17.52)	
Embryo stage			0.587						0.587
D2	3 (2.17)	11 (1.44)		/	/		3 (2.17)	11 (4.01)	
D3	132 (95.65)	258 (33.86)		/	/		132 (95.65)	258 (94.16)	
D5	3 (2.17)	5 (0.66)		/	/		3 (2.17)	5 (1.82)	

Data are mean ± standard deviation or N (% of response group). TE, transferrable embryos; Gn, gonadotropin; E2, estradiol; P4, progesterone; LH, luteinizing hormone; IVF, in vitro fertilization; ICSI, intracytoplasmic sperm injection; on HCG, on the day of human chorionic gonadotropin used.

In comparison 2, the usage of Gn, hormone levels on the day of HCG administration, type of ART, and the choice of stimulation protocol were different between two groups. Due to the difference of protocol choice, more dosage and days of Gn were used in TE group (P < 0.001).

In ET cycles, all patients underwent embryos implantation, number of oocytes (retrieved and MII oocytes, P = 0.004 and 0.001, respectively) and embryos (P < 0.001) differed significantly between pregnant and non-pregnant groups; hormone levels, Gn usage, embryo stage, and type of ART had no significant difference.

### Multivariate Logistic Regression and ROC Curve

To find which factors were associated with reproductive outcomes in women with DOR, univariate and multivariate logistic regression analyses were performed (**Table 3**).

**Table 3:** Factors associated with reproductive outcomes in women with DOR.

	Univariate			Multivariate		
	crude OR	95% CI	P value	adjusted OR	95% CI	P value
Model 1*						
Female age	0.096	0.95-1.00	0.978	0.981	0.95-1.01	0.191
Basal FSH (mIU/mL)	0.975	0.96-0.99	0.003	0.996	0.98-1.02	0.696
AFC	1.147	1.05-1.25	0.002	0.989	0.90-1.09	0.833
AMH (ng/mL)	11.848	6.21-22.62	<0.001	11.848	6.21-22.62	<0.001
**Model 2^#^**						
Type of infertility	1.676	1.08-2.60	0.021	0.995	0.54-1.83	0.988
No. of previous pregnancy	0.820	0.68-0.98	0.033	0.996	0.75-1.32	0.979
No. of previous deliveries	0.602	0.43-0.85	0.004	0.95	0.58-1.57	0.84
AFC	1.134	1.01-1.28	0.041	1.076	0.95-1.22	0.266
Female age						
<35	4.755	2.81-8.04	<0.001	4.755	2.81-8.04	<0.001
35-40	2.160	1.21-3.85	0.009	2.16	1.21-3.85	0.009
>40	reference			reference		
Male age						
<35	4.124	2.52-6.76	<0.001	2.067	0.93-4.62	0.077
35-40	1.504	0.83-2.73	0.181	1.049	0.52-2.10	0.893
>40	reference			reference		

OR, odds ratio; CI, confidence interval; AMH, anti-Müllerian hormone; FSH, follicle-stimulating hormone; AFC, antral follicle count.

*Model 1 included factors associated with TE; ^#^Model 2 included factors associated with clinical pregnancy in ET cycles.

**Figure 2:**
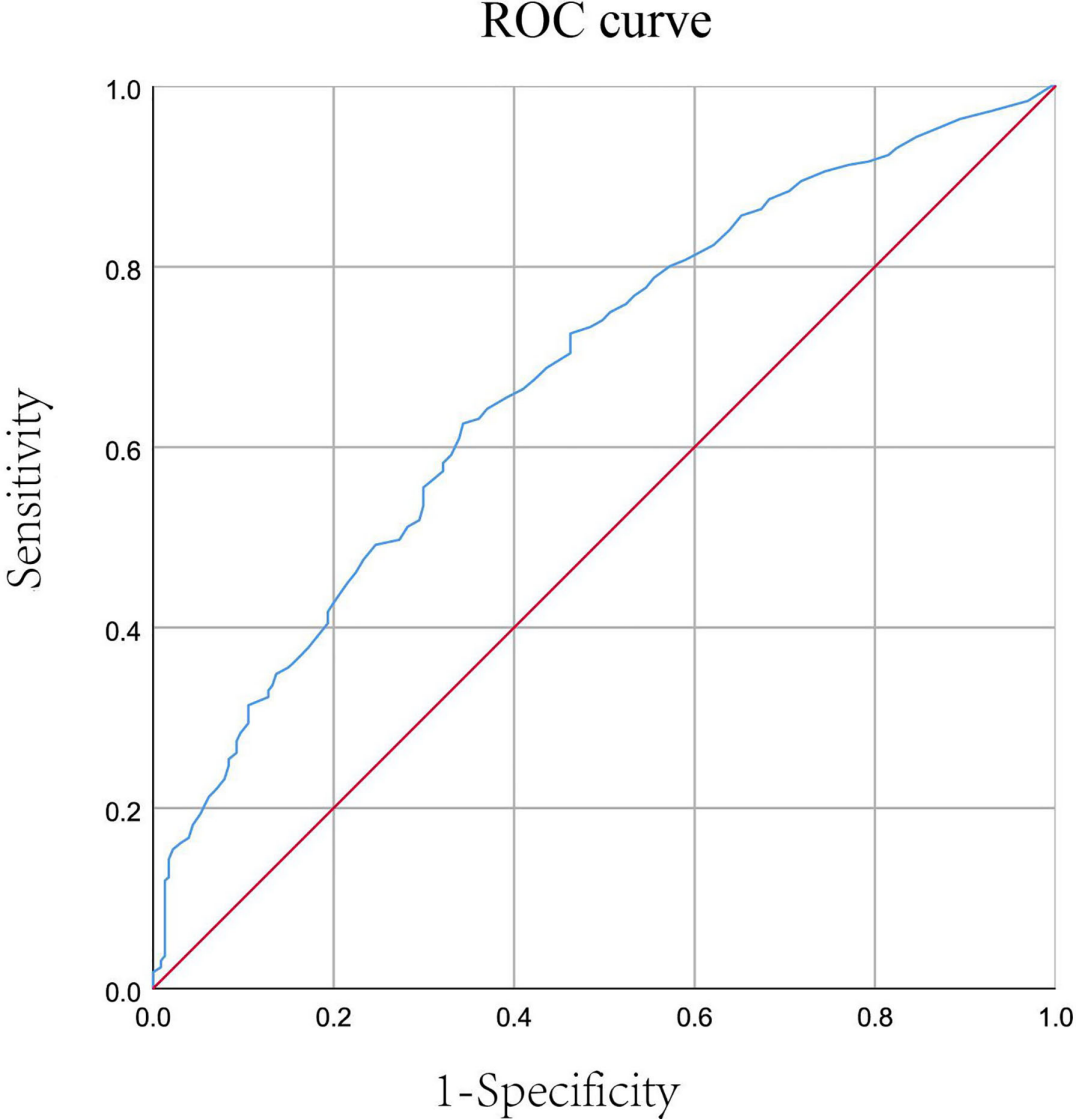
Receiver operating characteristics curve of the predictive utility of AMH for TE among women with DOR (area under the curve (AUC) = 0.679, 95% CI: 0.639-0.72, P < 0.001).

Model 1 included factors associated with TE, and results showed that AMH was an independent predictor of TE after adjusting for confounding factors (adjusted OR:11.848, 95% CI: 6.21-22.62, P< 0.001). Further ROC (receiver operating characteristic) analysis was performed and corresponding AUC (the area under the curve) was 0.679 (95% CI: 0.639-0.72, P< 0.001). Notably, AMH level of 0.355 had sensitivity of 62.6% and specificity of 65.6%. (**Figure 2**)

Model 2 included factors associated with clinical pregnancy in ET cycles. Female age was the only factor associated with clinical pregnancy in ET cycles and women under 35 years old were more likely to be pregnant compared to those older than 40 years old (adjusted OR:4.755, 95% CI: 2.81-8.04, P< 0.001).

## Discussion

In a woman’s life, the development of follicle pools begin when *in utero*. However, it begins to decline before the time of birth and continues to decline throughout the fertile years (14). Generally, the ovarian reserve drops sharply in the mid-40s, which is a normal physiological phenomenon. Some women, however, experience DOR long before the usual time, which causes infertility in their child-bearing years (15). DOR has multiple adverse implications for a woman’s health due to the change of ovarian hormones. A previous study demonstrated that it impairs renal function, increases the risk of cardiovascular disease and decreases bone mineral density (4). The most disastrous impact of DOR for a woman, however, may be infertility. With the development of ART, the number of patient visits is increasing rapidly, and about 31%of patients who go to reproductive centers for help have reduced ovarian reserve, and the incidence rises significantly with age (16). However, due to the long period and high cost of IVF/ICSI cycles, patients and families may have heavy burdens after failure, not only economically but also psychologically.

Therefore, we designed this study to find factors that affect fecundity in DOR women. Like previous reports (17, 18), patients were divided into groups based on if they were clinically pregnant in their first IVF/ICSI cycle, and age, AMH, and AFC were highly different between the two groups. Yet many patients (n = 139) had embryoes frozen because of elevated progesterone levels, uterus factor, or self-factors, and they were divided into the non-pregnant group. Biases may exist in the results above. It’s believed that women with higher AMH could have more eggs collect after COS (19). Next, to demonstrate AMH is also associated with the rate of embryo formation after retrieving oocytes, comparison 2 was constructed. Results showed AMH is an independent predictor of TE rate, and 0.355 ng/ml was a cutoff value for the prediction of TE. Last, we wondered if AMH can affect clinical pregnancy after embryo implantation, comparison 3 was made. Not surprisingly, female age is the only factor related to success in ET cycles.

### There Is No Best Protocol for DOR Patients Due to the Existence of Huge Individual Differences

Of the 900 women included, 762 women did not conceive. These patients were older and had much lower ovarian reserve according to FSH, AFC, and AMH compared to those who are pregnant. Considering baseline characteristics and poor ovarian response (POR) of these women, appropriate stimulation protocol was selected to avoid the adverse reactions of high-dose exogenous hormones and reduce the economic burden on patients. In COS cycles, patients in the non-pregnant group tended to use GnRH antagonist protocol and Follicular phase long-acting protocol. Natural protocol and mild stimulation protocol were in the non-pregnant group only. Correspondingly, women in non-pregnant group used much lower Gn and got fewer oocytes and embryos. Published data compared various protocols in DOR patients, GnRH agonist protocol and modified natural cycle were thought to be more effective than other protocols, since they could improve the quality of oocytes and probability of live birth of women with DOR (20, 21). The latest meta-analysis does not promote GnRH antagonist protocol for DOR patients because it correlates with higher cancellation rates and less pregnancies compared to agonist protocols (22). Ovarian stimulation protocol choice should be based on physical condition of DOR patients, which may be different each cycle. It is our opinion that there is no best protocol for this population because of the existence of huge individual differences.

### AMH Is an Independent Predictor of TE But Not of Clinical Pregnancy in ET Cycles

Produced by developing antral follicles in the ovaries and involved in the regulatory process of maturation of primordial follicles, AMH is considered an accurate biomarker to access ovarian reserve and ovarian response (15, 23). Recent studies tried to find out whether AMH had an association with reproductive outcomes, including rate of oocyte collected, clinical pregnancy, live birth, and miscarriage. A retrospective study reviewed 34,540 cycles with AMH<1 ng/ml and demonstrated serum AMH is highly correlated with cumulative live birth rates (CLBR) in women with DOR independent of age (24). Similarly, AMH was statistically differed between TE and no TE group in our study, and we identified 0.355 ng/ml as a cutoff value for the prediction of TE. Yet it had no correlation with clinical pregnancy in ET cycles, which means the AMH level was not associated with pregnancy rate in patients with implanted embryos. This result is consistent with a previous report (25). The ability of AMH on predicting the likelihood of IVF/ICSI success continues to be a subject of debate. Our study demonstrated that AMH is highly related to the oocyte acquired rate and TE rate but not to the clinical pregnancy rate in ET cycles. Large cohort studies are needed to discuss the relationship between them.

### Female Age Is a Risk Factor of Clinical Pregnancy in ET Cycles

Follicles in female ovarian apoptosis and the decrease with increasing of age means the capacity of fertility is dropping over time, therefore, age can largely determine whether conception can be successful. Studies have shown that in DOR patients, younger women have higher pregnancy rate and lower miscarriage rate compared to their older peers (8, 26, 27). Similar results were found in our study. Patients 35-40 years of age were 2.16 times more likely to getpregnant compared to those > 40 years old, and the number increased to 4.755 in patients < 35 years old. Researchers believe that DOR not only has adverse implications on oocyte quantity but also on quality (28). Moreover, data from our center investigated by Zhang et al. (29) showed that the aberration-related miscarriages among women with DOR were more frequent in patients older than 32 years old, and they demonstrated age is an independent risk factor for chromosomal abnormality after adjustment. For those who are diagnosed with DOR, younger women can have better reproductive outcomes with ART compared to older ones. This should be explained to patients so they can get a better understanding of their situation and get anxiety and stress released.

### Strength and Limitation

This study has some strengths. First, due to the rigorous definition of DOR, the homogeneity of the patients included was high, and possible confounding factors were removed. Second, we divided the patients into different groups step by step, and deeply explored the relevant factors related to the fecundity of DOR patients. This grouping method is conducive to controlling the influence of confounding factors.

There were also several limitations in our study. One is the nature of the retrospective study. Data from a single center also weakened the reliability. In addition, we only included the first IVF/ICSI cycle of these patients; CLBR were not analyzed. Conception, not live birth, was our main outcome, while live birth is crucial for accessing fecundity. Therefore, the conclusions from this study are not definitive but indicative, and these findings need to be confirmed by more prospective and multi-center studies.

## Conclusion

AMH is highly related to oocyte collection rate and TE rateand 0.355 ng/ml was a cutoff value for the prediction of TE. For DOR patients who had embryo transferred, AMH is not associated with clinical pregnancy while female age is an independent risk factor for it.

## Data Availability Statement

The original contributions presented in the study are included in the article/**Supplementary Material**. Further inquiries can be directed to the corresponding author.

## Ethics Statement

This study was performed under institutional review board approval of the First Affiliated Hospital of Zhengzhou University

## Author Contributions

LL and BS: designed study, analyzed data, and drafted the manuscript. FW and YZ: reviewed the manuscript. YS: Study conceptualization and review. All authors approved the final manuscript.

## Funding

This study was supported by Henan Province Medical Science and Technology Research (Key) Project (FDN-SBGJ202002049); Henan Province Medical Scienceand Technology Research Project (FDN-LHGJ20210353).

## Conflict of Interest

The authors declare that the research was conducted in the absence of any commercial or financial relationships that could be construed as a potential conflict of interest.

## Publisher’s Note

All claims expressed in this article are solely those of the authors and do not necessarily represent those of their affiliated organizations, or those of the publisher, the editors and the reviewers. Any product that may be evaluated in this article, or claim that may be made by its manufacturer, is not guaranteed or endorsed by the publisher.
